# Child health, inclusive education and development

**DOI:** 10.2471/BLT.22.288103

**Published:** 2022-06-06

**Authors:** Bolajoko O Olusanya, Nem Yun Boo, Olaf Kraus de Camargo, Mijna Hadders-Algra, Donald Wertlieb, Adrian C Davis, on behalf of the Global Research on Developmental Disabilities Collaborators (GRDDC)

**Affiliations:** a Center for Healthy Start Initiative, 286A Corporation Drive, Dolphin Estate, Ikoyi, Lagos, Nigeria.; b Department of Population Medicine, Universiti Tunku Abdul Rahman, Selangor, Malaysia.; c Center for Childhood Disability Research, McMaster University, Hamilton, Canada.; d Division of Developmental Neurology, University Medical Center Groningen, Groningen, Netherlands.; e Eliot-Pearson Department of Child Study & Human Development, Tufts University, Medford, United States of America.; f Department of Population Health, London School of Economics, London, England.

Since 2000, targeted reduction in mortality of children younger than 5 years has been the cornerstone of global child health policy. This policy was expanded with the sustainable development goals (SDGs) to include targeted reduction in neonatal mortality, which accounted for 41% (4 million/9.8 million) of child deaths in 2000.^1^^,^^2^ The United Nations (UN) Inter-agency Group for Child Mortality Estimation, led by the United Nations Children’s Fund (UNICEF), is entrusted with tracking progress on the neonatal and under-five mortality targets until 2030.^2^ According to the latest report of the inter-agency group published in 2021, neonatal mortality rate declined by 11% (from 19 to 17 deaths per 1000 livebirths) and under-five mortality rate declined by 14% (from 43 to 37 deaths per 1000 livebirths) in 2020 – 5 years after the launch of the SDGs. Many countries are on track to achieving the targets of 12 neonatal deaths and 25 child deaths per 1000 livebirths by 2030.^2^

## Children with disabilities

Saving the lives of children is a moral imperative for any society. However, the epidemiological transitions in low- and middle-income countries, accompanied by rapid population growth and increasing life expectancy, have raised ethical concerns about child health policies that are solely focused on child survival without adequate consideration for the development and well-being of the survivors with lifelong disabilities. More than 53 million children younger than 5 years and over 291 million children younger than 20 years are estimated to have disabilities from birth.^3^ An estimated 95% of children with a disability (younger than 5 years) reside in low- and middle-income countries.^3^ Globally, the likelihood of a surviving child having a disability such as hearing or vision loss, intellectual disability, epilepsy, autism or attention-deficit hyperactivity disorder is at least 10 times higher than that of dying before their fifth birthday.^3^ UNICEF has reported that compared with children without disabilities, children with disabilities are 42% less likely to have foundational reading and numeracy skills, 49% more likely to have never attended school, 47% more likely to drop out of primary school and 20% less likely to have expectations of a better life.^4^ Poor access to education places children with developmental disabilities at greater disadvantage in securing gainful employment. People with disabilities are frequently not considered potential members of the workforce. Anecdotal evidence shows that between 80% and 90% of people with disabilities of working age are estimated to be unemployed in low- and middle-income countries compared with between 50% and 70% in high-income countries.^5^ The developmental trajectory of children with disabilities – particularly in low- and middle-income countries – over the life course makes inclusive global child health, development and education policy more compelling.

## SDG framework for child health 

**Fig. 1:**
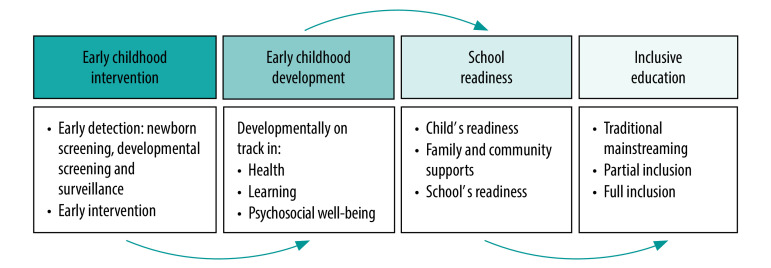
Pathway to inclusive child health, education and development

SDG 4 makes explicit provision for optimal early childhood development to ensure inclusive and equitable quality education and promote lifelong learning opportunities for all children, in addition to other targets.^1^ The policy of inclusion is enshrined in several treaties and legislative mandates including the World Declaration on Education for All (1990) and the UN Convention on the Rights of Persons with Disabilities (2006). The conceptual framework for early childhood development and care envisaged under SDG target 4.2 is geared towards facilitating school readiness for inclusive education for children with disabilities (Fig. 1). Child policies in high-income countries such as the Individuals with Disabilities Education Act in the United States of America exemplify this goal.^6^ The framework begins with the provision of early detection and intervention services for children in their first 5 years of life. These services typically include routine newborn screening for hearing impairment and vision loss followed by developmental screening and surveillance from age 6–9 months, to promptly detect and support children with developmental delays, disorders or disabilities.^7^ These services, which include providing physical and/or occupational therapy, hearing and/or visual aids, and speech therapy, help young children with disabilities to work towards meeting their developmental trajectory. Without this early and appropriate support, these children are unlikely to be ready for school enrolment. Therefore, the readiness of children with disabilities to attend school requires investment in these services, and ensuring that children receive necessary support from their family and community and that schools are ready to admit these children.^8^ Schools’ readiness to accept children with disabilities should include adequate number of such schools – where these children reside – physical facilities for children with special needs and specially trained teachers. Because of the vast differences in the nature, severity and complexity of disabilities in children, the implementation of inclusive education must be tailored to the specific needs of the child based on available resources and capacity. Children with severe or complex disabilities, for example, may begin their educational journey initially in a self-contained classroom (that is, partial inclusion in the regular school) and then gradually transit into the regular classroom (full inclusion). The common practice of placing children regardless of the nature of their disabilities in a regular classroom through traditional mainstreaming or integration without adequate support services is unlikely to facilitate effective inclusion and should be avoided.

## Health interlinked with education

Efforts to reduce avoidable neonatal and child mortality such as through improved immunization coverage and strengthening health systems for safer perinatal care should arguably have positive effects in improving health-care services and reducing the incidence of disabilities. For example, in 2019, neonatal conditions ranked as the fifth largest causes of death and the largest contributors to the disability-adjusted life years.^9^ However, interventions needed to prevent the leading causes of death often differ from those that can prevent or reduce morbidity and disability. Additionally, the health-care systems in low- and middle-income countries are underresourced to address the needs of children with disabilities.^10^ A major challenge in these countries is the absence of a globally funded initiative on early detection and intervention services to facilitate the introduction of national newborn and early childhood screening and rehabilitation programmes. This lack of investment is attributable to the overwhelming focus of global child health initiatives on survival, and the failure to recognize the critical role of early childhood development policies for the educational and vocational attainment of the surviving children with disabilities.^3^^,^^4^

The overarching message of the SDG target 4.2 is that health-related early childhood development must be geared towards equitable and inclusive education. Education is the basic building block of every society and the single best investment any country can make to build a healthy, prosperous and equitable society, as well as sustainable development. Access to inclusive and quality education enhances opportunities for gainful employment and social inclusion. Gainfully employed people with disabilities make significant contributions to the sustainable development of their society rather than constitute an economic and social burden. Without the concerted efforts of relevant UN agencies and national governments to ensure that the beneficiaries of global child survival initiatives (of children with disabilities) are placed on a trajectory for optimal early childhood development in readiness for inclusive education, it is unlikely that the commitments made under the SDGs will be fulfilled. Moreover, without a well-coordinated longer-term vision for global health and educational agenda, the prevailing socioeconomic inequalities between high-income and low- and middle-income countries will increase.

## Data for monitoring progress 

Data drive global policy initiatives and investment. We consider that UNICEF, the lead agency for the Inter-agency Group for Child Mortality Estimation, sole custodian of early childhood development under the SDGs and a key player in global child health, should ensure that estimates of children younger than 5 years who have disabilities are routinely included in the yearly State of the World’s Children report. Additional indicators for tracking progress such as the proportion of children with disabilities receiving early detection and intervention services, those receiving organized learning, and those completing primary education should be considered. The exclusion of children younger than 24 months in the revised indicator for monitoring early childhood development among children younger than 5 years should be addressed as it undermines the inclusivity principle of leaving no one behind in the SDGs. Estimates from the World Health Organization and World Bank’s Model Disability Survey and data on specific diagnostic entities from the Global Burden of Disease Study will continue to serve as valuable complements to the conventional UNICEF data derived from parent-reported child functioning. This integration is consistent with the International Classification of Functioning, Disability and Health, which emphasizes the interrelationship between deficits in body structure (health conditions) and functional limitations.^11^ Such comprehensive data on child functioning and specific underlying health conditions or impairments will also guide the development and provision of requisite support services, thereby enhancing the value of these services for children with disabilities.

Inclusion in global child health is not limited to education. Child health and well-being must also be considered within the context of emergent global threats such as the coronavirus disease 2019 and future pandemics, forced migration and climate change.^12^ A disability-inclusive global child health policy will ensure that the beneficiaries of the substantial global investments in child survival with lifelong disabilities are adequately and appropriately supported in their early crucial years of human development for inclusive education and development. 
